# Progress Rate Analysis of Evolution Strategies on the Rastrigin Function: First Results

**DOI:** 10.1007/978-3-031-14721-0_35

**Published:** 2022-08-15

**Authors:** Amir Omeradzic, Hans-Georg Beyer

**Affiliations:** Research Center Business Informatics, Vorarlberg University of Applied Sciences, Hochschulstraße 1, 6850 Dornbirn, Austria

**Keywords:** Evolution Strategies, Rastrigin function, Progress rate analysis, Global optimization

## Abstract

A first order progress rate is derived for the intermediate multi-recombinative Evolution Strategy (*μ*/*μ_I_*, *λ*)-ES on the highly multimodal Rastrigin test function. The progress is derived within a linearized model applying the method of so-called noisy order statistics. To this end, the mutation-induced variance of the Rastrigin function is determined. The obtained progress approximation is compared to simulations and yields strengths and limitations depending on mutation strength and distance to the optimizer. Furthermore, the progress is iterated using the dynamical systems approach and compared to averaged optimization runs. The property of global convergence within given approximation is discussed. As an outlook, the need of an improved first order progress rate as well as the extension to higher order progress including positional fluctuations is explained.

## 1 Introduction

Evolution Strategies (ES) [12,13] are well-recognized Evolutionary Algorithms suited for real-valued non-linear optimization. State-of-the-art ES such as the CMA-ES [8] or its simplification [5] are also well-suited for locating global optimizers in highly multimodal fitness landscapes. While the CMA-ES was originally mainly intended for non-differentiable optimization problems, but yet regarded as a locally acting strategy, it was already in [7] observed that using a large population size can make the ES a strategy that is able to locate the global optimizer among a huge number of local optima. This is a surprising observation when considering the ES as a strategy that acts mainly local in the search space following some kind of gradient or natural gradient [3,6,11]. As one can easily check using standard (highly) multimodal test functions such as Rastrigin, Ackley, and Griewank to name a few, this ES property is not intimately related to the covariance matrix adaptation (CMA) ES which generates non-isotropic correlated mutations, but can also be found in (*μ/μ_I_*, λ)-ES with *isotropic* mutations. Therefore, if one wants to understand the underlying working principles how the ES locates the global optimizer, the analysis of the (*μ/μ_I_*, λ)-ES should be the starting point.

The question regarding why and when optimization algorithms − originally designed for local search − are able to locate global optima has gained attention in the last few years. A recurring idea comes from relaxation procedures that transform the original multimodal optimization problem into a convex optimization problem called Gaussian continuation [9]. Gaussian continuation is nothing else but a convolution of the original optimization problem with a Gaussian kernel. As has been shown in [10], using the right Gaussian, Rastrigin-like functions can be transformed into a convex optimization problem, thus making it accessible to gradient following strategies. However, this raises the question how to perform the convolution efficiently. One road followed in [14] uses high-order Gauss-Hermite integration in conjunction with a gradient descent strategy yielding surprisingly good results. The other road coming to mind is approximating the convolution by Gaussian sampling. This resembles the procedure ES do: starting from a parental state, offspring are generated by Gaussian mutations. The problem is, however, that in order to get a reliable gradient, a huge number of samples, i.e. offspring in ES must be generated in order to get reliable convolution results. The number of offspring needed to get reliable estimates seems much larger than the offspring population size needed in ES experiments conducted in [7] showing approximately a linear relation between problem dimension *N* and population size for the Rastrigin function. Therefore, understanding the ES performance from viewpoint of Gaussian relaxation does not seem to help much.

The approach followed in this paper will incorporate two main concepts, namely a progress rate analysis as well as its application within the so-called evolution equations modeling the transition dynamics of the ES [2]. The progress rate measure yields the expected positional change in search space between two generations depending on location, strategy and test function parameters. Aiming to investigate and understand the dynamics of globally converging ES runs, the progress rate is an essential quantity to model the expected evolution dynamics over many generations.

This paper provides first results of a scientific program that aims at an analysis of the performance of the (*μ/μ_I_*, λ)-ES on Rastrigin’s test function based on a first order progress rate. After a short introduction of the (*μ/μ_I_*, λ)-ES, the *N*-dimensional first order progress will be defined and an approximation will be derived resulting in a closed form expression. The predictive power and its limitations will be checked by one-generation experiments. The progress rate will then be used to simulate the ES dynamics on Rastrigin using difference equations. This simulation will be compared with real runs of the (*μ/μ_I_*, λ)-ES. In a concluding section a summary of the results and outlook of the future research will be given.

## 2 Rastrigin Function and Local Quality Change

The real-valued minimization problem defined for an *N*-dimensional search vector **y** = (*y*_1_, …, *y_N_*) is performed on the Rastrigin test function *f* given by

(1) $$f(y)=\sum_{i=1}^{N}f_{i}\left(y_{i}\right)=\sum_{i=1}^{N}y_{i}^{2}+A-A\cos\left(\alpha y_{i}\right),$$

with *A* denoting the oscillation amplitude and *α* = 2*π* the corresponding frequency. The quadratic term with superimposed oscillations yields a finite number of local minima *M* for each dimension *i*, such that the overall number of minima scales exponentially as *M^N^* posing a highly multimodal minimization problem. The global optimizer is at **ŷ** = **0**.

For the progress rate analysis in Sect. 4 the local quality function *Q*_**y**_(**x**) at **y** due to mutation vector **x** = (*x*_1_, …, *x_N_*) is needed. In order to reuse results from noisy progress rate theory it will be formulated for the *maximization* case of *F*(**y**) = −*f*(**y**) with *F_i_*(*y_i_*) = −*f_i_*(*y_i_*), such that local quality change yields

(2) $$Q_{\text{y}}(\text{x})=F(\text{y}+\text{x})-F(\text{y})=f(\text{y})-f(\text{y}+\text{x}).$$

*Q*_**y**_(**x**) can be evaluated for each component *i* independently giving

(3) $$Q_{y}(\text{x})=\sum_{i=1}^{N}Q_{i}\left(x_{i}\right)=\sum_{i=1}^{N}f_{i}\left(y_{i}\right)-f_{i}\left(y_{i}+x_{i}\right)$$

(4) $$=\sum_{i=1}^{N}-\left(x_{i}^{2}+2y_{i}x_{i}+A\cos\left(\alpha y_{i}\right)\left(1-\cos\left(\alpha x_{i}\right)\right)+A\sin\left(\alpha y_{i}\right)\sin\left(\alpha x_{i}\right)\right).$$

A closed form solution of the progress rate appears to be obtainable only for a linearized expression of *Q_i_*(*x_i_*). A first approach taken in this paper is based on a Taylor expansion for the mutation *x_i_* and discarding higher order terms

(5) $$Q_{i}\left(x_{i}\right)=F_{i}\left(y_{i}+x_{i}\right)-F_{i}\left(y_{i}\right)=\frac{\partial F_{i}}{\partial y_{i}}x_{i}+O\left(x_{i}^{2}\right)$$

(6) $$\approx \left(-2y_{i}-\alpha A\sin\left(\alpha y_{i}\right)\right)x_{i}=:-f_{i}^{\prime }x_{i},$$

using the following derivative terms

(7) $$k_{i}=2y_{i}\;\text{and}\;d_{i}=\alpha A\sin\left(\alpha y_{i}\right),\;\text{such}\;\mathrm{that}\;\frac{\partial f_{i}}{\partial y_{i}}=f_{i}^{\prime }=k_{i}+d_{i}.$$

A second approach is to consider only the linear term of Eq. (4) and neglect all non-linear terms denoted by *δ*(*x_i_*) according to

(8) $$Q_{i}\left(x_{i}\right)=-2y_{i}x_{i}-x_{i}^{2}-A\cos\left(\alpha y_{i}\right)\left(1-\cos\left(\alpha x_{i}\right)\right)-A\sin\left(\alpha y_{i}\right)\sin\left(\alpha x_{i}\right)$$

(9) $$=-2y_{i}x_{i}+\delta \left(x_{i}\right)\approx -2y_{i}x_{i}=-k_{i}x_{i}.$$

The linearization using $f_{i}^{\prime }$ is a local approximation of the function incorporating oscillation parameters *A* and *α*. Using only *k_i_* (setting *d_i_* = 0) discards oscillations by approximating the quadratic term via $k_{i}=\partial \left(y_{i}^{2}\right)/\partial y_{i}=2y_{i}$ with negative sign due to maximization. Both approximations will be evaluated later.

## 3 The (*μ*/*μ_I_*, λ)-ES with Normalized Mutations

The Evolution Strategy under investigation consists of a population of *μ* parents and λ offspring (*μ* < λ) per generation *g*. Algorithm 1 is presented below and offspring variables are denoted with overset “∼”.

Population variation is achieved by applying an isotropic normally distributed mutation **x** ∼ *σ𝒩*(0, **1**) with strength *σ* to the parent recombinant in Lines 6 and 7. The recombinant is obtained using intermediate recombination of all *μ* parents equally weighted in Line 11. Selection of the *m* = 1, …, *μ* best search vectors **y**_*m*;λ_ (out of λ) according to their fitness is performed in Line 10.

Note that the ES in Algorithm 1 operates under constant normalized mutation σ* in Lines 3 and 12 using the spherical normalization

(10) $$\sigma^{*}=\frac{\sigma^{\left(g\right)}N}{\left||y^{\left(g\right)}|\right|}=\frac{\sigma^{\left(g\right)}N}{R^{\left(g\right)}}.$$

This property ensures global convergence of the algorithm as the mutation strength *σ*^(*g*)^ decreases if and only if the residual distance ║**y**^(*g*)^║ = *R*^(*g*)^ decreases. While σ* is not known during black-box optimizations, it is used here to investigate the dynamical behavior of the ES using the first order progress rate approach to be developed in this paper. Incorporating self-adaptation of *σ* or cumulative step-size adaptation remains for future research.

Algorithm 1 (*μ/μ_I_*, λ)-ES with constant σ*

1: *g* ← 0

2: **y**^(0)^ ← **y**^(init)^

3: *σ*^(0)^ ← σ* ║**y**^(0)^║ /*N*

4: **repeat**

5:     **for** *l* = 1, …, λ **do**

6:         ${\tilde{x}}_{l}\leftarrow \sigma^{\left(g\right)}{𝒩}_{l}(0,1)$

7:         ${\tilde{y}}_{l}\leftarrow y^{\left(g\right)}+{\tilde{x}}_{l}$

8:         ${\tilde{f}}_{l}\leftarrow f\left({\tilde{y}}_{l}\right)$

9:     **end for**

10:    $\left({\tilde{y}}_{1;\lambda },\ldots ,{\tilde{y}}_{\mu ;\lambda }\right)\leftarrow \text{sort}\;(\tilde{y}\;\text{w}.\text{r}.\text{t}.\;\text{ascending}\;\tilde{f})$

11:    $y^{\left(g+1\right)}\leftarrow \frac{1}{\mu }\sum_{m=1}^{\mu }{\tilde{y}}_{m;\lambda }$

12:    *σ*^(*g*+1)^ ← σ* ║**y**^(*g*+1)^║/*N*

13:    *g* ← *g* + 1

14: **until** termination criterion

## 4 Progress Rate

### 4.1 Definition

Having introduced the Evolution Strategy, we are interested in the expected one-generation progress of the optimization on the Rastrigin function (1) before investigating the dynamics over multiple generations.

A first order progress rate *φ_i_* for the *i*-th component between two generations *g* → *g* + 1 can be defined as the expectation value over the positional difference of the parental components

(11) $$\varphi_{i}=\text{E}\left[y_{i}^{\left(g\right)}-y_{i}^{\left(g+1\right)}|\sigma^{\left(g\right)},y^{\left(g\right)}\right]=y_{i}^{\left(g\right)}-\text{E}\left[y_{i}^{\left(g+1\right)}|\sigma^{\left(g\right)},y^{\left(g\right)}\right],$$

given mutation strength *σ*^(*g*)^ and the position **y**^(*g*)^. First, an expression for **y**^(*g*+1)^ is needed, see Algorithm 1, Line 11. It is the result of mutation, selection and recombination of the *m* = 1, …, *μ* offspring vectors yielding the highest fitness, such that $y^{\left(g+1\right)}=\frac{1}{\mu }\sum_{m=1}^{\mu }{\tilde{y}}_{m;\lambda }=\frac{1}{\mu }\sum_{m=1}^{\mu }{\left(y^{\left(g\right)}+x\right)}_{m;\lambda }$. Considering the *i*-th component, noting that **y**^(*g*)^ is the same for all offspring and setting (**x**_*m*;λ_)_*i*_ = *x*_*m*;λ_ one has

(12) $$y_{i}^{\left(g+1\right)}=\frac{1}{\mu }\sum_{m=1}^{\mu }\left(y_{i}^{\left(g\right)}+x_{m;\lambda }\right)=y_{i}^{\left(g\right)}+\frac{1}{\mu }\sum_{m=1}^{\mu }x_{m;\lambda }.$$

Taking the expectation $\text{E}\left[y_{i}^{\left(g+1\right)}\right]$, setting *x* = *σz* = *σ𝒩*(0, 1) and inserting the expression back into (11) yields

(13) $$\varphi_{i}=-\frac{1}{\mu }\text{E}\left[\sum_{m=1}^{\mu }x_{m;\lambda }|\sigma^{\left(g\right)},y^{\left(g\right)}\right]=-\frac{\sigma }{\mu }\text{E}\left[\sum_{m=1}^{\mu }z_{m;\lambda }|\sigma^{\left(g\right)},y^{\left(g\right)}\right].$$

Therefore progress can be evaluated by averaging over the expectations of *μ* selected mutation contributions. In principle this task can be solved by deriving the induced order statistic density *p_m;λ_* for the *m*-th best individual and subsequently solving the integration over the *i*-th component

(14) $$\varphi_{i}=-\frac{1}{\mu }\sum_{m=1}^{\mu }\int_{-\infty }^{\infty }x_{i}\;p_{m;\lambda }\left(x_{i}|\sigma^{\left(g\right)},y^{\left(g\right)}\right)\text{d}x_{i}.$$

However, the task of computing expectations of sums of order statistics under noise disturbance has already been discussed and solved by Arnold in [1]. Therefore the problem of Eq. (13) will be reformulated in order to apply the solutions provided by Arnold.

### 4.2 Expectations of Sums of Noisy Order Statistics

Let *z* be a random variate with density *p_z_*(*z*) and zero mean. The density is expanded into a Gram-Charlier series by means of its cumulants *κ_i_* (*i* ≥ 1) according to [1, p. 138, D.15]

(15) $$p_{z}(z)=\frac{1}{\sqrt{2\pi \kappa_{2}}}{\text{e}}^{-\frac{z^{2}}{2\kappa_{2}}}\left(1+\frac{\gamma_{1}}{6}{\text{He}}_{3}\left(\frac{z}{\sqrt{\kappa_{2}}}\right)+\frac{\gamma_{2}}{24}{\text{He}}_{4}\left(\frac{z}{\sqrt{\kappa_{2}}}\right)+\ldots \right),$$

with expectation *κ*_1_ = 0, variance *κ*_2_, skewness $\gamma_{1}=\kappa_{3}/\kappa_{2}^{3/2}$, excess $\gamma_{2}=\kappa_{4}/\kappa_{2}^{2}$ (higher order terms not shown) and He_*k*_ denoting the *k*-th order probabilist’s Hermite polynomials. For the problem at hand, see Eq. (13), the mutation variate *z* ∼ *𝒩*(0, 1) with *κ*_2_ = 1 and *κ_i_* = 0 for *i* ≠ 2 yielding a standard normal density.

Furthermore, let $\epsilon ∼𝒩\left(0,\sigma_{\epsilon }^{2}\right)$ model additive noise disturbance, such that resulting observed values are *v* = *z* + *ϵ*. Selection of the *m*-th largest out of λ values yields

(16) $$v_{m;\lambda }=(z+𝒩(0,\sigma_{\epsilon }^{2}){)}_{m;\lambda },$$

and the distribution of selected source terms *z_m;λ_* follows a noisy order statistic with density *p_m;λ_*. Given this definition and a linear relation between *z_m;λ_* and *v_m;λ_* the method of Arnold is applicable.

In our case the *i*-th mutation component *x_m;λ_* of Eq. (13) is related to selection via the quality change defined in Eq. (3). Maximizing the fitness *F_i_*(*y_i_* + *x_i_*) conforms to maximizing quality *Q_i_*(*x_i_*) with *F_i_*(*y_i_*) being a constant offset.

Aiming at an expression of form (16) and starting with (3), we first isolate component *Q_i_* from the remaining *N* − 1 components denoted by Σ_*j*≠*i*_ *Q_j_*. Then, approximations are applied to both terms yielding

(17) $$Q_{y}(\text{x})=Q_{i}\left(x_{i}\right)+\sum_{j\neq i}Q_{j}\left(x_{j}\right)$$

(18) $$\approx -f_{i}^{\prime }x_{i}+𝒩\left(E_{i},D_{i}^{2}\right),$$

with linearization (6) applied to *Q_i_*(*x_i_*). Additionally, $\sum_{j\neq i}\;Q_{j}≃𝒩\left(E_{i},D_{i}^{2}\right)$, as the sum of independent random variables asymptotically approaches a normal distribution in the limit *N* → ∞ due to the Central Limit Theorem. This is ensured by Lyapunov’s condition provided that there are no dominating components within the sum due to largely different values of *y_j_*. The corresponding Rastrigin quality variance $D_{i}^{2}=\text{Var}\left[\sum_{j\neq i}\;Q_{j}(x_{j})\right]$ is calculated in the supplementary material (https://github.com/omam-evo/paper/blob/main/ppsn22/PPSN22_OB22.pdf). As the expectation *E_i_* = E[Σ_*j*≠*i*_ *Q_j_*(*x_j_*)] is only an offset to *Q*_**y**_(**x**) it has no influence on the selection and its calculation can be dropped.

Using *x_i_* = *σz_i_* and $f_{i}^{\prime }=\mathrm{sgn}\left(f_{i}^{\prime }\right)\left|f_{i}^{\prime }\right|$, expression (18) is reformulated as

(19) $$Q_{y}(\text{x})=-\mathrm{sgn}\left(f_{i}^{\prime }\right)\left|f_{i}^{\prime }\right|\sigma z_{i}+E_{i}+𝒩\left(0,D_{i}^{2}\right)$$

(20) $$\frac{Q_{\text{y}}(\text{x})-E_{i}}{\left|f_{i}^{\prime }\right|\sigma }=\mathrm{sgn}\left(-f_{i}^{\prime }\right)z_{i}+𝒩\left(0,\frac{D_{i}^{2}}{{\left(f_{i}^{\prime }\sigma \right)}^{2}}\right).$$

The decomposition using sign function and absolute value is needed for correct ordering of selected values w.r.t. *z_i_* in (20).

Given result (20), one can define the linearly transformed quality measure $v_{i}:=\left(Q_{y}(x)-E_{i}\right)/\left|f_{i}^{\prime }\right|\sigma$ and noise variance $\sigma_{\epsilon }^{2}:={\left(D_{i}/f_{i}^{\prime }\sigma \right)}^{2}$, such that the selection of mutation component sgn $\left(-f_{i}^{\prime }\right)z_{i}$ is disturbed by a noise term due to the remaining *N* − 1 components. A relation of the form (16) is obtained up to the sign function.

In [1] Arnold calculated the expected value of arbitrary sums *S_P_* of products of noisy ordered variates containing *ν* factors per summand

(21) $$S_{P}=\sum_{\left\{n_{1},\ldots ,n_{\nu }\right\}}z_{n_{1};\lambda }^{p_{1}}\cdots z_{n_{\nu };\lambda }^{p_{\nu }},$$

with random variate *z* introduced in Eqs. (15) and (16). The vector *P* = (*p*_1_, …, *p_ν_*) denotes the positive exponents and distinct summation indices are denoted by the set {*n*_1_, …, *n_ν_*}. The generic result for the expectation of (21) is provided in [1, p. 142, D.28] and was adapted to account for the sign difference between (16) and (20) resulting in possible exchanged ordering. Performing simple substitutions in Arnold’s calculations in [1] and recalling that in our case *γ*_1_ = *γ*_2_ = 0, the expected value yields

(22) $$\text{E}\left[S_{P}\right]=\mathrm{sgn}{\left(-f_{i}^{\prime }\right)}^{\left||P_{1}|\right|}{\sqrt{\kappa_{2}}}^{\left||P_{1}|\right|}\frac{\mu !}{(\mu -\nu )!}\sum_{n=0}^{\nu }\sum_{k\geq 0}\zeta_{n,0}^{\left(P\right)}(k)h_{\mu ,\lambda }^{\nu -n,k}.$$

Note that expression (22) deviates from Arnold’s formula only in the sign in front of $\sqrt{\kappa_{2}}$. The coefficients $\zeta_{n,0}^{\left(P\right)}(k)$ are defined in terms of a noise coefficient *a* according to

(23) $$a=\sqrt{\frac{\kappa_{2}}{\kappa_{2}+\sigma_{\epsilon }^{2}}}\;\text{ with }\;\zeta_{n,0}^{\left(P\right)}(k)=\text{Polynomial}(a),$$

for which tabulated results are presented in [1, p. 141]. The coefficients $h_{\mu ,\lambda }^{i,k}$ are numerically obtainable solving

(24) $$h_{\mu ,\lambda }^{i,k}=\frac{\lambda -\mu }{\sqrt{2\pi }}\left(\begin{array}{l} \lambda \\ \mu \end{array}\right)\int_{-\infty }^{\infty }\text{H}{\text{e}}_{k}(x){\text{e}}^{-\frac{1}{2}x^{2}}[\phi (x){]}^{i}[\Phi (x){]}^{\lambda -\mu -1}[1-\Phi (x){]}^{\mu -i}\text{d}x.$$

Now we are in the position to calculate expectation (13) using (22). Since *z* ~ *𝒩*(0,1), it holds *κ*_2_ = 1. Identifying *P* = (1), ║*P*║_1_ = 1 and *ν* = 1 yields

(25) $$\begin{array}{l} \text{E}\left[\sum_{m=1}^{\mu }z_{m;\lambda }\right]=\mathrm{sgn}\left(-f_{i}^{\prime }\right)\frac{\mu !}{(\mu -1)!}\sum_{n=0}^{1}\sum_{k\geq 0}\zeta_{n,0}^{\left(1\right)}(k)h_{\mu ,\lambda }^{1-n,k}\\ \;=\mathrm{sgn}\left(-f_{i}^{\prime }\right)\mu \zeta_{0,0}^{\left(1\right)}(0)h_{\mu ,\lambda }^{1,0}=-\mathrm{sgn}\left(f_{i}^{\prime }\right)\mu ac_{\mu /\mu ,\lambda }, \end{array}$$

with $\zeta_{1,0}^{\left(1\right)}(k)=0$ for any *k*, and $\zeta_{0,0}^{\left(1\right)}(k)\neq 0$ only for *k* = 0 yielding *a*. The expression $h_{\mu ,\lambda }^{1,0}$ is equivalent to the progress coefficient definition *c*_*μ/μ,λ*_ [2, p. 216]. Inserting (25) back into (13), using $a=\sqrt{1/(1+{\left(D_{i}/f_{i}^{\prime }\sigma \right)}^{2})}=\left|f_{i}^{\prime }\right|\sigma /\sqrt{{\left(f_{i}^{\prime }\sigma \right)}^{2}+D_{i}^{2}}$ with the requirement *a* > 0, and noting that $f_{i}^{\prime }=\mathrm{sgn}\left(f_{i}^{\prime }\right)\left|f_{i}^{\prime }\right|$ one finally obtains for the *i*-th component first order progress rate

(26) $$\varphi_{i}(\sigma ,y)=c_{\mu /\mu ,\lambda }\frac{f_{i}^{\prime }\left(y_{i}\right)\sigma^{2}}{\sqrt{{\left(f_{i}^{\prime }\left(y_{i}\right)\sigma \right)}^{2}+D_{i}^{2}\left(\sigma ,{\left(y\right)}_{j\neq i}\right)}}.$$

The population dependency is given by progress coefficient *c*_*μ/μ*_,λ. The fitness dependent parameters are contained in $f_{i}^{\prime }$, see (7), and in $D_{i}^{2}$ calculated in the supplementary material (https://github.com/omam-evo/paper/blob/main/ppsn22/PPSN22_OB22.pdf). For better readability the derivative $f_{i}^{\prime }$ and variance $D_{i}^{2}$ are not inserted into (26). An exemplary evaluation of $D_{i}^{2}$ as a function of the residual distance *R* using normalization (10) is also shown in the supplementary material.

### 4.3 Comparison of Simulation and Approximation

**Fig. 1:**
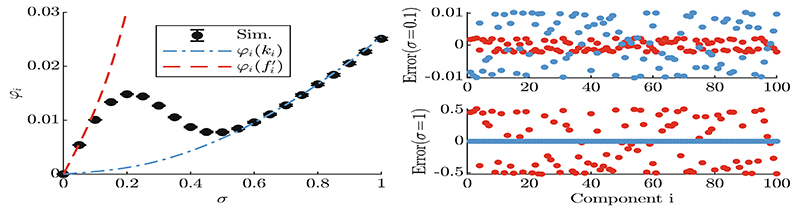
One-generation experiments with (150/150, 300)-ES, *N* = 100, *A* = 10 are performed and quantity (11) is measured averaging over 10^5^ runs. Left: *φ_i_* over *σ* for *i* = 2 at position *y*_2_ ≈ 1.19, where **y** was chosen randomly such that ║**y**║ = *R* = 10. Right: error measure *φ_i_* − *φ*_*i*,sim_ between (26) and simulation for *i* = 1, …, *N* evaluated at *σ* = {0.1, 1}. The colors are set according to the legend. (Color figure online)

Figure 1 shows an experimentally obtained progress rate compared to the result of (26). Due to large *N* one exemplary *φ_i_*-graph is shown on the left, and corresponding *i* = 1, …, *N* errors are shown on the right.

The left plot shows the progress rate over a *σ*-range of [0, 1]. This magnitude was chosen in order to study the oscillation, as the frequency *α* = 2*π*. The initial position was chosen randomly to be on the sphere surface *R* = 10.

The red dashed curve uses $f_{i}^{\prime }$ as linearization, while the blue dash-dotted curve assumes $f_{i}^{\prime }=k_{i}$ (with *d_i_* = 0), see also (7). As $f_{i}^{\prime }$ approximates the quality change locally, agreement for the progress is given only for very small mutations *σ*. For larger *σ* very large deviation may occur, depending on the local derivative.

The blue curve *φ_i_*(*k_i_*) neglects the oscillation (*d_i_* = 0) and therefore follows the progress of the quadratic function $f(y)=\sum_{i}\;y_{i}^{2}$ for large *σ* with very good agreement. Due to a *linearized* form of *Q_i_*(*x_i_*) in (6) neither approximation can reproduce the oscillation for moderately large *σ*.

To verify the approximation quality, the error between (26) and simulation is displayed on the right side of Fig. 1 for all *i* = 1, …, *N*. It was done for small *σ* = 0.1 and large *σ* = 1. The deviations are very similar in magnitude for all *i*, given randomly chosen *y_i_*. Note that for *σ* = 1 the red points show very large errors compared to blue, which was expected.

**Fig. 2:**
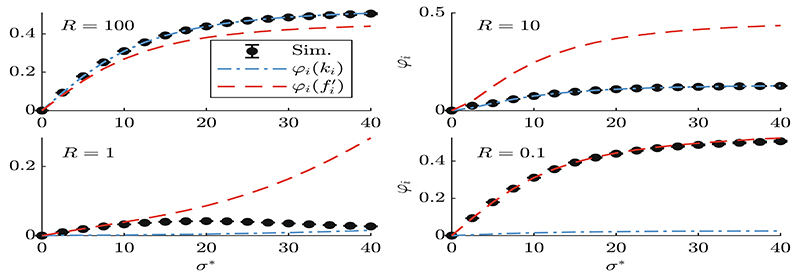
One-generation progress *φ_i_* (*i* = 2) over normalized mutation σ* for (150/150, 300)-ES, *N* = 100, *A* = 1 and *R* = {100, 10, 1, 0.1}. Simulations are averaged over 10^5^ runs. These experiments are preliminary investigations related to the dynamics shown in Fig. 3 with σ* = 30. Given a constant σ* the approximation quality varies over different magnitudes of *R*.

**Fig. 3:**
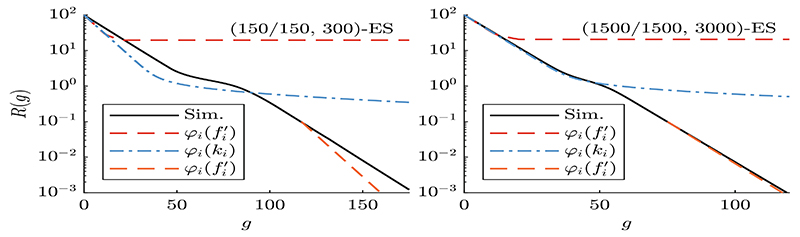
Comparing average of 100 optimization runs of Algorithm 1 (black, solid) with iterated dynamics from Eq. (27) under constant σ* = 30 for *A* = 1 and *N* = 100. Large populations sizes are chosen to ensure global convergence (left: *μ* = 150; right: *μ* = 1500; constant *μ/λ* = 0.5). Iteration using progress (26) is performed for both $f_{i}^{\prime }=k_{i}+d_{i}$ (red/orange dashed) and $f_{i}^{\prime }\left(d_{i}=0\right)=k_{i}$ (blue dash-dotted) using Equations (27) and (28). The orange dashed iteration was initialized with *R*^(0)^ = 0.1 and translated to the corresponding position of the simulation for easier comparison. The evaluation of quality variance $D_{i}^{2}(R)$ is shown in the supplementary material (https://github.com/omam-evo/paper/blob/main/ppsn22/PPSN22_OB22.pdf). (Color figure online)

Figure 2 shows the progress rate *φ_i_* over σ*, for *i* = 2 as in Fig. 1, with **y** randomly on the surface radii *R* = {100, 10, 1, 0.1}. Using σ* the mutation *σ* is normalized by the residual distance *R* with spherical normalization (10). Far from the origin with *R* = {100, 10} the quadratic terms are dominating giving better results using *φ_i_*(*k_i_*). Reaching *R* = 1 local minima are more relevant and mixed results are obtained with $\varphi_{i}\left(f_{i}^{\prime }\right)$ better for smaller σ* and *φ_i_*(*k_i_*) for larger σ*. Within the global attractor *R* = 0.1 the local structure dominates and $\varphi_{i}\left(f_{i}^{\prime }\right)$ yields better results. These observations will be relevant analyzing the dynamics in Fig. 3 where both approximations show strengths and weaknesses.

## 5 Evolution Dynamics

As we are interested in the dynamical behavior of the ES, averaged real optimization runs from Algorithm 1 will be compared to the iterated dynamics using progress result (26) by applying the dynamical systems approach [2]. Neglecting fluctuations, i.e., $y_{i}^{\left(g+1\right)}=\text{E}\left[y_{i}^{\left(g+1\right)}|\sigma^{\left(g\right)},y^{\left(g\right)}\right]$ the mean value dynamics for the mapping $y_{i}^{\left(g\right)}\to y_{i}^{\left(g+1\right)}$ immediately follows from (11) giving

(27) $$y_{i}^{\left(g+1\right)}=y_{i}^{\left(g\right)}-\varphi_{i}\left(\sigma^{\left(g\right)},y^{\left(g\right)}\right).$$

The control scheme of *σ*^(*g*)^ was introduced in Eq. (10) and yields simply

(28) $$\sigma^{\left(g\right)}=\sigma^{*}||y^{\left(g\right)}||/N.$$

Equations (27) and (28) describe a deterministic iteration in search space and rescaling of mutations according to the residual distance. For a convergence analysis, we are interested in the dynamics of *R*^(*g*)^ = ║**y**^(*g*)^║ rather than the actual position values **y**^(*g*)^. Hence in Fig. 3 the *R*^(*g*)^-dynamics of the conducted experiments is shown.

In Fig. 3, all runs of Algorithm 1 exhibit global convergence with the black line showing the average. The left and right plots differ by population size. Iteration *φ_i_*(*k_i_*), blue dash-dotted curve, also converges globally, though very slowly and therefore not shown entirely. The convergence behavior of iteration $\varphi_{i}\left(f_{i}^{\prime }\right)$, red and orange dashed curves, strongly depends on the initialization and is discussed below.

Three phases can be observed for the simulation. It shows linear convergence at first being followed by a slow-down due to local attractors. Reaching the global attractor the convergence speed increases again. Iteration *φ_i_*(*k_i_*) is able to model the first two phases to some degree. Within the global attractor the slope information *d_i_* is missing such that the progress is largely underestimated.

Iteration $\varphi_{i}\left(f_{i}^{\prime }\right)$ converges first, but yields a stationary state with *R^st^* ≈ 20 when the progress *φ_i_* becomes dominated by derivative term *d_i_*. Starting from *R*^(0)^ = 10^2^ the stationary $y_{i}^{st}$ are either fixed or alternating between coordinates depending on *σ, D_i_, k_i_*, and *d_i_*. This effect is due to attraction of local minima and due to the deterministic iteration disregarding fluctuations. It occurs also with varying initial positions. Initialized at *R*^(0)^ = 10^−1^ orange iteration $\varphi_{i}\left(f_{i}^{\prime }\right)$ is globally converging.

It turns out that the splitting point of the two approximations in Fig. 3 occurs at a distance *R* to the global optimizer where the ES approaches the attractor region of the “first” local minima. For the model parameters considered in the experiment this is at about *R* ≈ 28.2 − the distance of the farest local minimizer to the global optimizer (obtained by numerical analysis).

Plots in Fig. 3 differ by population size. The convergence speed, i.e. the slopes, show better agreement for large populations, which can be attributed to the fluctuations neglected in (27). Investigations on unimodal funtions Sphere [2] and Ellipsoid [4] have shown that progress is decreased by fluctuations due to a loss-term scaling with 1/*μ*, which agrees with Fig. 3. On the left the iterated progress is faster due to neglected but present fluctuations, while on the right better agreement is observed due to insignificant fluctuations. These observations will be investigated in future research.

## 6 Summary and Outlook

A first order progress rate *φ_i_* was derived for the (*μ/μ_I_*, λ)-ES by means of noisy order statistics in (26) on the Rastrigin function (1). To this end, the mutation induced variance of the quality change $D_{i}^{2}$ is needed. Starting from (4) a derivation yielding $D_{i}^{2}$ has been presented in the supplementary material. Furthermore, the approximation quality of *φ_i_* was investigated using Rastrigin and quadratic derivatives $f_{i}^{\prime }$ and *k_i_*, respectively, by comparing with one-generation experiments.

Linearization $f_{i}^{\prime }$ shows good agreement for small-scale mutations, but very large deviations for large mutations. Conversely, linearization *k_i_* yields significantly better results for large mutations as the quadratic fitness term dominates. A progress rate modeling the transition between the regimes is yet to be determined. First numerical investigations of (14) including all terms of (4) indicate that nonlinear terms are needed for a better progress rate model, which is an open challenge and part of future research.

The obtained progress rate was used to investigate the dynamics by iterating (27) using (28) and comparing with ES runs. Iteration via $f_{i}^{\prime }$ only converges globally if initialized close to the optimizer, since local attraction is strongly dominating. Dynamics via *k_i_* converges globally independent of initialization, but the observed rate matches only for the initial phase and for very large populations. This confirms the need for a higher order progress rate modeling the effect of fluctuations, especially when function evaluations are expensive and small populations must be used. Additionally, an advanced progress rate formula is needed combining effects of global and local attraction to model all three phases of the dynamics correctly.

The investigations done so far are a first step towards a full dynamical analysis of the ES on the multimodal Rastrigin function. Future investigations must also include the complete dynamical modeling of the mutation strength control. One aim is the tuning of mutation control parameters such that the global convergence probability is increased while still maintaining search efficiency. Our final goal will be the theoretical analysis of the full evolutionary process yielding also recommendations regarding the choice of the minimal population size needed to converge to the global optimizer with high probability.
